# Supplementary material for: Intracranial Efficacy of Atezolizumab, Bevacizumab, Carboplatin, and Paclitaxel in Real-World Patients with Non-Small-Cell Lung Cancer and EGFR or ALK Alterations
Source: Cancers (Basel). 2024 Mar 22;16(7):1249. doi: 10.3390/cancers16071249 (PMC11011096; doi:10.3390/cancers16071249)
Supplement: Supplementary file 1 [file cancers-16-01249-s001.zip › cancers-2910914-supplementary.pdf]

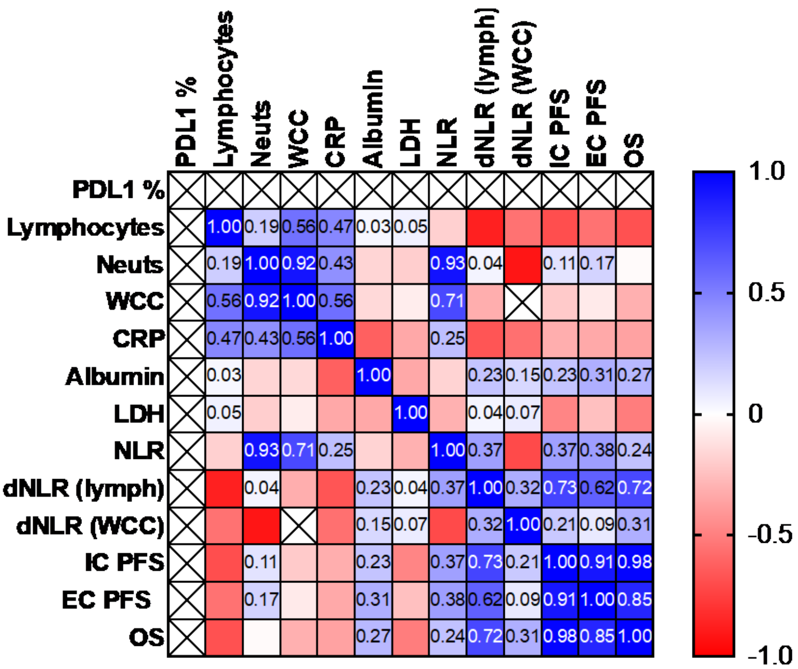

Supplementary Figure S1. Pearson R-matrix of patients with CNS involvement and PDL1<1%. dNLR (derived Neutrophil Lymphocyte Ratio) was calculated using lymphocyte count (lymph) and total white cell count (WCC). R values are plotted in each box.

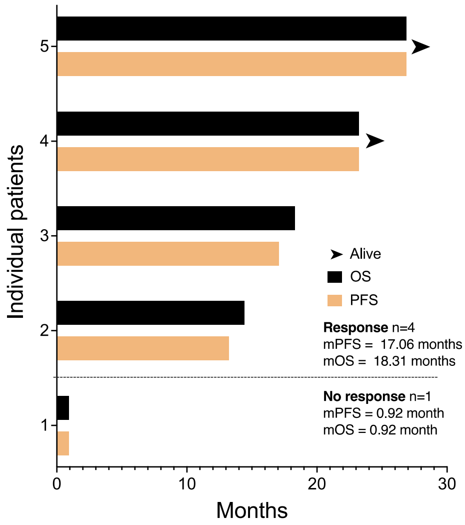

Supplementary Figure S2. Clinical outcomes plotted for individual patients divided according to radiological response. mOS; median Overall Survival, mPFS; median Progression Free Survival
